# IMPROVEMENTS IN HEALTH-RELATED QUALITY OF LIFE FOR BINGOCIZE CLINICAL TRIAL PARTICIPANTS

**DOI:** 10.1093/geroni/igac059.2389

**Published:** 2022-12-20

**Authors:** Emily Smith, Matthew Shake, Callie Hamm, Jason Crandall

**Affiliations:** Western Kentucky University, Louisville, Kentucky, United States; Western Kentucky University, Bowling Green, Kentucky, United States; Western Kentucky University, Louisville, Kentucky, United States; Western Kentucky University, Bowling Green, Kentucky, United States

## Abstract

With the number of older adults increasing rapidly, researchers have increasingly focused on designing interventions to improve health-related quality of life (HRQOL) in older adulthood. However, many interventions struggle with adherence because older adults often perceive them as unenjoyable, condescending, or painful. Here, we report results from a clinical trial of Bingocize®, a community-based “in vivo” exercise and health education intervention for older adults, to determine if participation impacts participants’ HRQOL. One-hundred and forty-three older adults ages 60+ were randomly assigned to one of four conditions that contrasted exercise and health education to non-intervention control groups. All conditions were matched on social engagement and met in group sessions twice weekly for 12 weeks. The CDC HRQOL measure was administered before and after the intervention. Session adherence was >90% across all sessions. Results from 2 (Time: Pre/Post) x 4 (Condition: Bingo-only Control vs. Bingo+Health Education vs. Bingo+Exercise vs. Bingo+Health Education+Exercise) ANOVAs found that all participants reported better sleep quality, reduced pain, and increased energy after completing the program (p-values <.05). Results from 2 (Time: Pre/Post) x 2 (Exercise/No Exercise) ANOVAs revealed interactions showing that exercise participants experienced greater decreases in days with anxiety and physically unhealthy days as compared to non-exercise participants (p-values <.05). These findings suggest that elements of Bingocize can contribute to improvements in older adults’ mental and physical quality of life. The current research can help researchers and professionals further elucidate which intervention mechanisms play a role in determining older adults’ health-related quality of life.

